# Supplementary material for: Vaccination with a Replication-Dead Murine Gammaherpesvirus Lacking Viral Pathogenesis Genes Inhibits WT Virus Infection
Source: Viruses. 2024 Dec 17;16(12):1930. doi: 10.3390/v16121930 (PMC11680341; doi:10.3390/v16121930)
Supplement: Supplementary file 1 [file viruses-16-01930-s001.zip › viruses-3355852-Supplemental Table1-revised.pdf]

Table S1. Primers, gBlocks and antibodies used in this study

Mutant MHV68 generation	
**Mutation**	**gBlock[a]**
ORF50 locus<br>50_STOP_FS_gBLK_1	AGAAACCAGAAGGTGAGGTTTAATGCCAAAGTCCATAACAGGCATCCATGTGGGTACATA**TAG**TCTC<br>ACCACCTGATC**T**AAATATGCCATTGA**T**AAGAGTTGTCTA**G**ACCACAGACAGGCTGTTTCTTAGGGATA<br>ACAGGGTAATCGATTTATTCAACAAAGCCACGTTGTGTCTCAAAATCTCTGATGTTACATTGCACAAGA<br>TAAAAATATATCATCATGAACAATAAAACTGTCTGCTTACATAAACAGTAATACAAGGGGTGTTATGA<br>GCCATATTCAACGGGAAACGTCTTGCTCGAGGCCGCGATTAAATTCCAACATGGATGCTGATTTATATG<br>GGTATAAATGGGCTCGCGATAATGTCGGGCAATCAGGTGCGACAATCTATCGATTGTATGGGAAGCCC<br>GATGCGCCAGAGTTGTTTCTGAAACATGGCAAAGGTAGCGTTGCCAATGATGTTACAGATGAGATGGT<br>CAGACTAAACTGGCTGACGGAATTTATGCCTCTTCCGACCATCAAGCATTTTATCCGTACTCCTGATGA<br>TGCATGGTTACTCACCACTG
ORF50 locus<br>50_STOP_FS_gBLK_2	CTCCTGATGATGCATGGTTACTCACCACTGCGATCCCCGGGAAAACAGCATTCCAGGTATTAGAAGAA<br>TATCCTGATTCAGGTGAAAATATTGTTGATGCGCTGGCAGTGTTCCTGCGCCGGTTGCATTCGATTCCTG<br>TTTGTAATTGTCCTTTTAACAGCGATCGCGTATTTCGTCTCGCTCAGGCGCAATCACGAATGAATAACG<br>GTTTGGTTGATGCGAGTGATTTTGATGACGAGCGTAATGGCTGGCCTGTTGAACAAGTCTGGAAAGAA<br>ATGCATAAGCTTTTGCCATTCTCACCGGATTCAGTCGTCACTCATGGTGATTTCTCACTTGATAACCTTA<br>TTTTTGACGAGGGGAAATTAATAGGTTGTATTGATGTTGGACGAGTCGGAATCGCAGACCGATACCAG<br>GATCTTGCCATCCTATGGAACTGCCTCGGTGAGTTTTCTCCTTCATTACAGAAACGGCTTTTTCAAAAAT<br>ATGGTATTGATAATCCTGATATGAATAAATTGCAGTTTCATTTGATGCTCGATGAGTTTTTCTAATCAGA<br>ATTGGTTAATTGGTTGTAACACTGGCGGCATCCATGTGGGTACATA**TAG**TCTCACCACCTGATC**T**AAAT<br>ATGCCATTGA**T**AAGAGTTGTCTA**G**ACCACAGACAGGCTGTTTCTGACTGCCCCATGTTCGGGGGTACA<br>GCTACCTCAACCTCTG
M1-M4 deletion<br>M1_4DEL gBLK_1	CCTTTATTGTAAGGGTACTCTCATCACCAATGTAAATTAATATGTAGCAAACTTTGGTGTGGGAGTCCT<br>ACCCCTTTTGCTCCACAGGCCGCCCACGACCATCTAGCCTCCCACCAAAACAAGAACAGTTGCAGCTT<br>TTGCTGTTTTCCTTAATATTTATCTTTGTGGTGTTCAGTCAGGCCTTGCTCTCAGTTTCTATGCCCCCAGG<br>CTGGTTTCAAACACTGGTCAGAAGGCTATCTTTCTTGGTGGTTTCACTTCTAAACATGGGCCATTAAAA<br>GGGAGGGAATTGGCATCATTGAGCAGCGGCGACCTAGGGATAACAGGGTAATCGATTTATTCAACAA<br>AGCCACGTTGTGTCTCAAAATCTCTGATGTTACATTGCACAAGATAAAAATATATCATCATGAACAAT<br>AAAACTGTCTGCTTACATAAACAGTAATACAAGGGGTGTTATGAGCCATATTCAACGGGAAACGTCTT<br>GCTCGAGGCCGCGATTAAATTCCAACATGGATGCTGATTTATATGGGTATAAATGGGCTCGCGATAAT<br>GTCGGGCAATCAGGTGCGACAATCTATCGATTGTATGGGAAGCCCGATGCGCCAGAGTTGTTTCTGAA

	ACATGGCAAAGGTAGCGTTGCCAATGATGTTACAGATGAGATGGTCAGACTAAACTGGCTGACGGAA<br>TTTATGCCTCTTCCGACCATCAAGCATTTTATCCGTACTCCTGATGATGCATGGTTACTCACCACTG
M1-M4 deletion<br>M1_4DEL gBLK_2	CTCCTGATGATGCATGGTTACTCACCACTGCGATCCCCGGGAAAACAGCATTCCAGGTATTAGAAGAA<br>TATCCTGATTCAGGTGAAAATATTGTTGATGCGCTGGCAGTGTTCCTGCGCCGGTTGCATTCGATTCCTG<br>TTTGTAATTGTCCTTTTAACAGCGATCGCGTATTTCGTCTCGCTCAGGCGCAATCACGAATGAATAACG<br>GTTTGGTTGATGCGAGTGATTTTGATGACGAGCGTAATGGCTGGCCTGTTGAACAAGTCTGGAAAGAA<br>ATGCATAAGCTTTTGCCATTCTCACCGGATTCAGTCGTCACTCATGGTGATTTCTCACTTGATAACCTTA<br>TTTTTGACGAGGGGAAATTAATAGGTTGTATTGATGTTGGACGAGTCGGAATCGCAGACCGATACCAG<br>GATCTTGCCATCCTATGGAACTGCCTCGGTGAGTTTTCTCCTTCATTACAGAAACGGCTTTTTCAAAAAT<br>ATGGTATTGATAATCCTGATATGAATAAATTGCAGTTTCATTTGATGCTCGATGAGTTTTTCTAATCAGA<br>ATTGGTTAATTGGTTGTAACACTGGCTTCTAAACATGGGCCATTAAAAGGGAGGGAATTGGCATCATT<br>GAGCAGCGGCGACCTTACATTCATCTGGGAATATGGTATTGAGATTTATGACTTTCTAGAATAACTGTA<br>CCCTGTTAAGTTTCAATTCCTTGTGGCCCTACCCCGAATCTCTATTAAAGGGTTAATAAAAATTACTCTC<br>AACAAAATCATGGCCAACTTCCACTTTTTCTGCGCAGTATTGGTGGGGATTGTGGGTGTAAATGGTGAC<br>AACATGTGCCACCATCTTCCTCAAATGCCCACACTAACATCTCCTAAATTTACACCACCAGTCAAAAG<br>TGGCACCACCCTGCAGCTCCGCTGTAGGCCAGGGTTCACACCAGGCGCA
**Mutation**	**Amplification Primers**
RDV-50.stop<br>50_STOP_FS_FOR	5′AGAAACCAGAAGGTGAGGTTTAATG
RDV-50.stop<br>50_STOP_FS_REV	5′CAGAGGTTGAGGTAGCTGTACCC
RDV-50.stopΔM1-M4<br>M1-4_DEL_FOR	5′-CACCAATGTAAATTAATATGTAGC
RDV-50.stopΔM1-M4<br>M1-4_DEL_REV	5′-AACCCTGGCCTACAGCGGAGCTGC
**Mutation**	**Sanger Sequencing Primers**
ORF50 locus<br>50_FSS_SEQ_FOR	5′ACAAATTTTACACAGCACCTGAAGC
ORF50 locus<br>50_FSS_SEQ_REV	5′ATGCCTCAACTTCTCTGGATATG

M1-M4 deletion<br>M1-4_SEQ_FOR	5′-CACCAATGTAAATTAATATGTAGC
M1-M4 deletion<br>M1-4_SEQ_REV	5'-GGTGGTGGTGCTCATGTCTGACG

[a]Bold letters - **TAG** and two **T's** were added, then a **G** was substituted for a T to obtain a *Xba*I site for screening which sufficiently disrupts the reading frame. Underlined-frameshift region
