# Supplementary material for: Vaccination with a Replication-Dead Murine Gammaherpesvirus Lacking Viral Pathogenesis Genes Inhibits WT Virus Infection
Source: Viruses. 2024 Dec 17;16(12):1930. doi: 10.3390/v16121930 (PMC11680341; doi:10.3390/v16121930)
Supplement: Supplementary file 1 [file viruses-16-01930-s001.zip › viruses-3355852-supplementary-figure-revised.pdf]

Supplemental Figure Legends

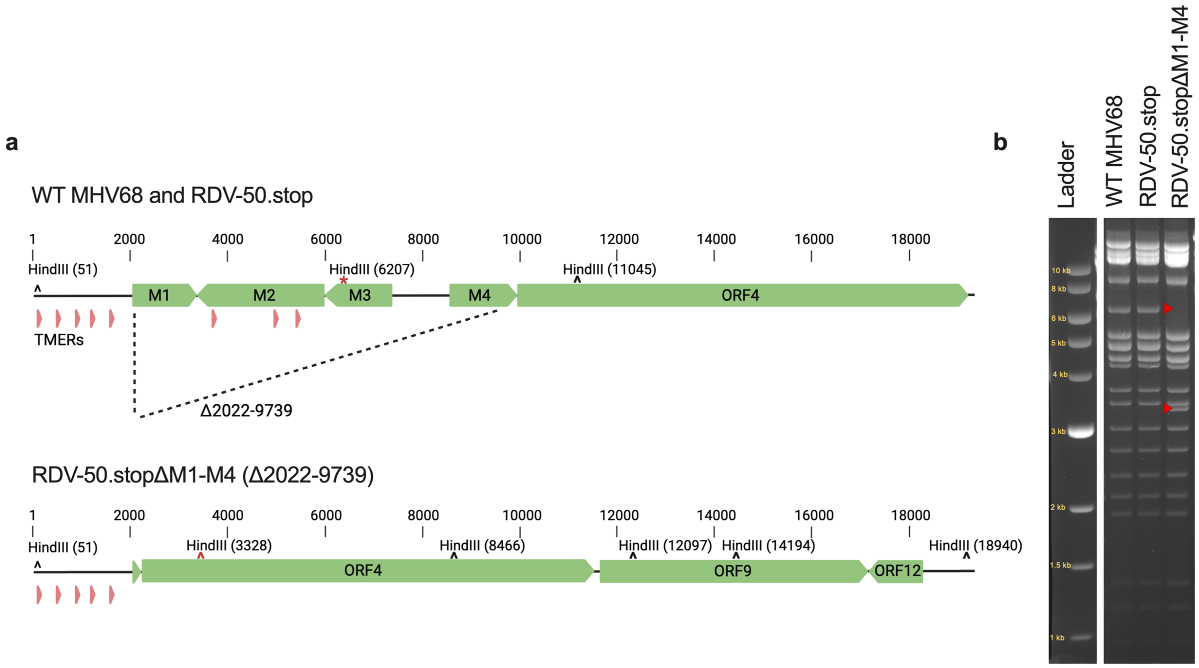

Supplementary Figure 1 Generation of recombinant RDV-50.stopΔM1-M4 MHV68 upon deletion of unique M gene locus. (a) Schematic describing the left end of WT MHV68 and the parental RDV-50.stop. M1- M4 genes and TMERs 6-8 (nucleotides 2022 to 9739) were deleted from RDV-50.stop to generate MHV68 RDV-50.stopΔM1-M4. '*' and '^' denote HindIII digestion sites. '*' indicates the site lost with the deletion of 2022 to 9739 bp. Figure made with BioRender. **(b)** In the HindIII restriction digest, WT MHV68 and RDV-50.stop has the expected 6156 bp fragment, while the RDV-50.stopΔM1-M4 genome has the expected loss of the 6156 bp fragment and gain of a 3277 bp fragment, indicated by red arrows.

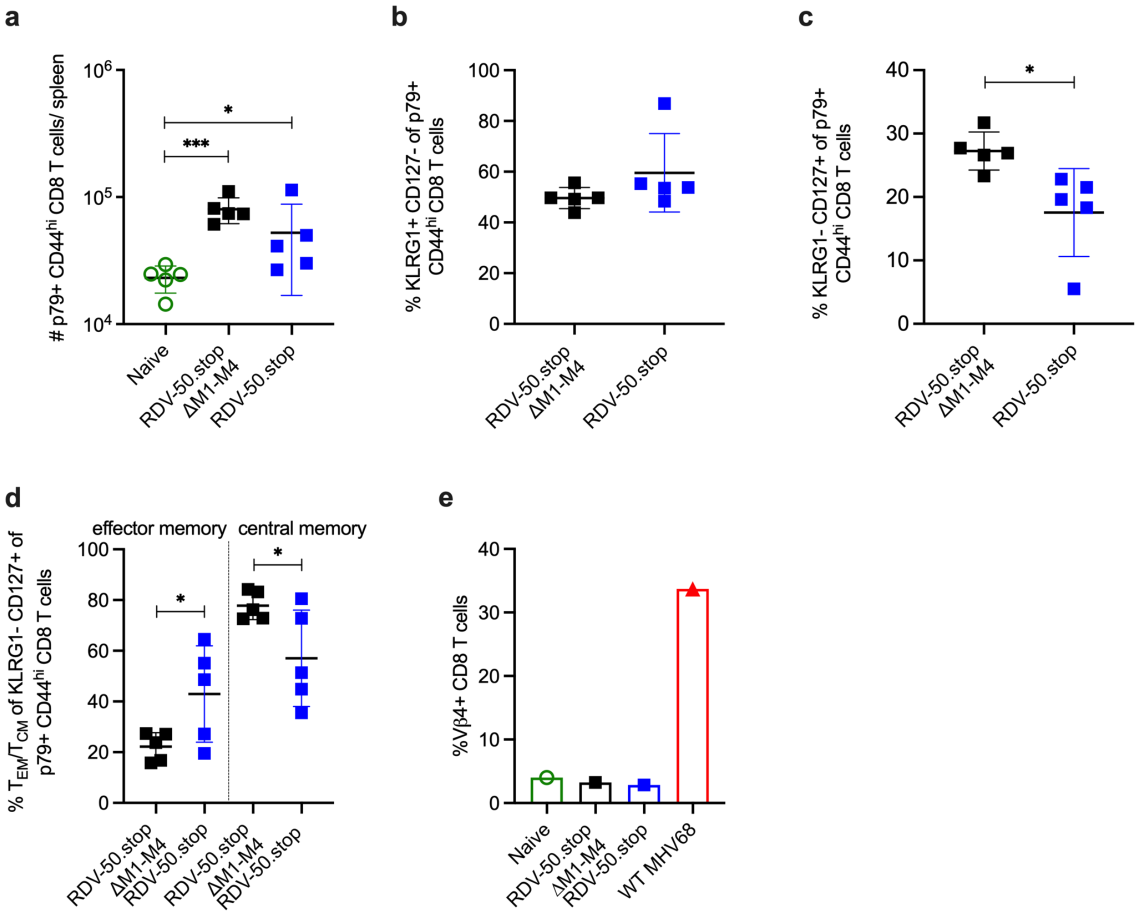

Supplementary Figure 2 RDV-50.stopΔM1-M4 generates virus-specific CD8 T cell responses but does not induce M1 driven Vβ4+ CD8 T cells upon a prime-boost regimen in C57BL/6 mice. C57BL/6 mice were either sham-vaccinated or prime-boost vaccinated IP with 1x10^6 PFU RDV-50.stopΔM1-M4 or RDV-50.stop. Naïve mice were age-matched, non-vaccinated controls. **(a)** Total p79-tetramer+ CD8 T cells per spleen of individual mice at d28 post-boost. Percentage of p79-dextramer+ CD8 T cells with markers of **(b)** short-lived effector cell (SLEC, KLRG1+CD127-) and **(c)** memory precursor effector cell subsets (MPEC, KLRG1-CD127+). **(d)** MPECs were further delineated into CD62L- effector and CD62L+ central MPECs for p79- dextramer+ CD8 T cells. **(e)** Percentage of Vβ4+ CD8 T cells in pooled spleens of mice with RDV-50.stop or RDV-50.stopΔM-M4 vaccination or infection with WT MHV68. For **(a-d)**, symbols represent individual mice, (N=5), and for **(e)** symbols represent pooled spleens from 5 mice.; bars and whiskers are mean +/- SD. *, p<0.05; **, p<0.01; ***, p<0.001 in Sidak's multiple comparisons test of one-way ANOVA **(a)**; in two-tailed unpaired t test **(b-d)** between the indicated groups.

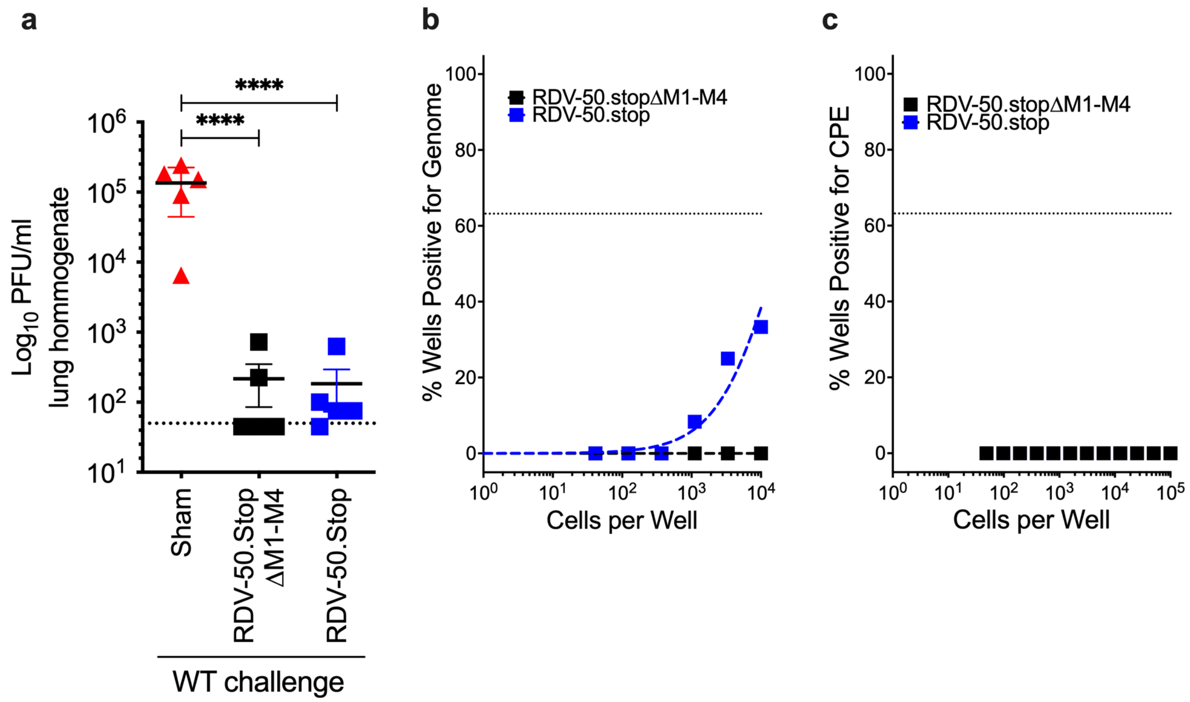

Supplementary Figure 3 Vaccination with RDV-50.stopΔM1-M4 reduces acute replication, latency and reactivation in C57BL/6 mice. Mice were either sham-vaccinated or prime-boost vaccinated IP with $1x10^6$ PFU of RDV-50.stopΔM1-M4 or RDV-50.stop followed by IP challenge with $5x10^3$ PFU of WT MHV68 at d28 post-boost. **(a)** Acute replication at d7 post-challenge determined by measuring infectious particles per ml lung homogenate. (N=5 mice per experiment); bars and whiskers represent mean +/- SD. ****, p<0.0001 in Sidak's multiple comparisons test of one-way ANOVA between the indicated groups. **(b)** The frequency of latency determined by limiting dilution nested PCR of intact splenocytes for the viral genome at day d28 post-boost. **(c)** The frequency of explant reactivation determined by limiting dilution coculture of intact viable splenocytes on a monolayer of primary MEFs d28 post-boost. Disrupted splenocytes plated in parallel did not detect preformed infectious virus in the vaccinated animals. For **(b-c),** symbols represent pool of five individual mice per experiment.

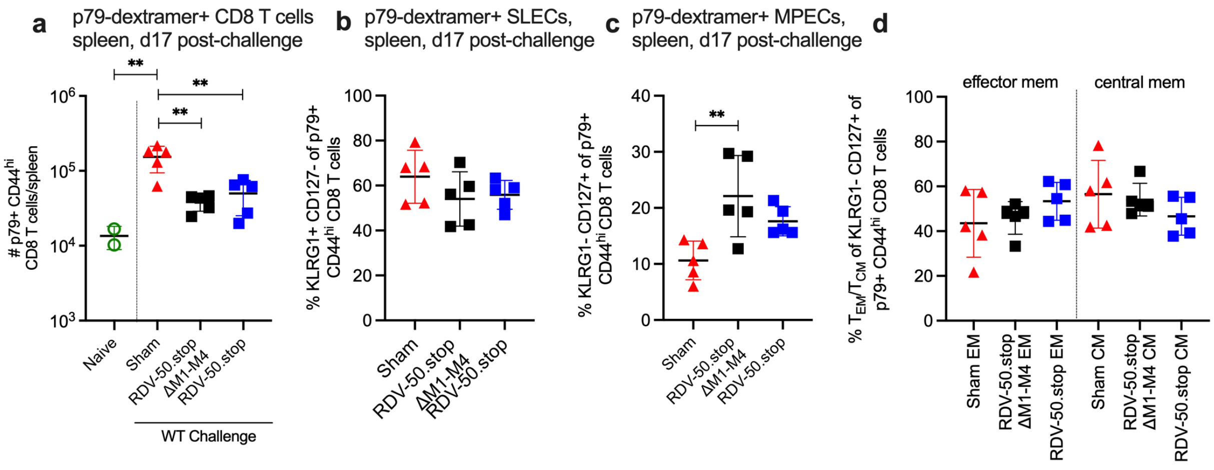

Supplementary Figure 4 Evaluation of T cell response to MHV68 in the spleens of vaccinated mice at seventeen days post-challenge with WT virus. C57BL/6 mice were either sham-vaccinated or prime-boost vaccinated IP with $1x10^6$ PFU RDV-50.stopΔM1-M4 or RDV-50.stop followed by IN challenge with $1x10^3$ PFU WT MHV68 at d28 post-boost and analyzed d17 post-challenge. **(a)** Total p79-dextramer+ CD8 T cells per spleen of individual mice with the indicated vaccination regimen. Percentage of p79-dextramer+ CD8 T cells with markers of **(b)** short-lived effector cell (SLEC, $KLRG1^+CD127^-$) and **(c)** memory precursor effector cell subsets (MPEC, $KLRG1^-CD127^+$). **(d)** MPECs were further delineated into $CD62L^-$ effector and $CD62L^+$ central MPECs for p79-dextramer+ CD8 T cells. For each graph, symbols represent individual mice, (N=3-5); bars and whiskers are mean +/- SD. **, p<0.01 in Sidak's multiple comparisons test of one-way ANOVA between the indicated groups.
